# CircRNA-miRNA-VEGFA: an important pathway to regulate cancer pathogenesis

**DOI:** 10.3389/fphar.2023.1049742

**Published:** 2023-05-10

**Authors:** Lei Zhang, Yuan Zhang, Xin Li, Huijuan Gao, Xiatian Chen, Peifeng Li

**Affiliations:** Institute for Translational Medicine, The Affiliated Hospital of Qingdao University, Qingdao University, Qingdao, China

**Keywords:** circRNA, VEGFA, cancers, pathogenesis, functional mechanisms

## Abstract

Cancers, especially malignant tumors, contribute to high global mortality rates, resulting in great economic burden to society. Many factors are associated with cancer pathogenesis, including vascular endothelial growth factor-A (VEGFA) and circular RNAs (circRNA). VEGFA is a pivotal regulator of vascular development such as angiogenesis, which is an important process in cancer development. CircRNAs have covalently closed structures, making them highly stable. CircRNAs are widely distributed and participate in many physiological and pathological processes, including modulating cancer pathogenesis. CircRNAs act as transcriptional regulators of parental genes, microRNA (miRNA)/RNA binding protein (RBP) sponges, protein templates. CircRNAs mainly function via binding to miRNAs. CircRNAs have been shown to influence different diseases such as coronary artery diseases and cancers by regulating VEGFA levels via binding to miRNAs. In this paper, we explored the origin and functional pathways of VEGFA, reviewed the current understanding of circRNA properties and action mechanisms, and summarized the role of circRNAs in regulating VEGFA during cancer pathogenesis.

## Introduction

Cancer is defined as the result of abnormal cells dividing in an uncontrolled way that forms a tumor mass, which can be benign or malignant. Malignant tumors are difficult to treat, resulting in high mortality rates. Cancer compromises human health and results in an economic burden for patients, healthcare systems, and countries. Thus, scientists have been exploring the mechanisms underlying cancer incidence and development (Liu et al., 2022c; Zhou et al., 2022). Vascular endothelial growth factor-A (VEGFA) is an important factor affecting cancer pathogenesis. VEGFA belongs to a large protein family that includes VEGFB, VEGFC, VEGFD, VEGFE (virally encoded only), and the placental growth factor. VEGFs are pivotal regulators of vascular development. Among all the VEGF proteins, VEGFA plays a major role and may sometimes be referred to as VEGF directly. VEGFA regulates most endothelial responses, modulates angiogenesis and participates in the pathogenesis of various angiogenesis-related diseases, including cancers.

CircRNA is one type of noncoding RNA with a cyclic structure (Ao and Liu, 2022). CircRNAs are highly stable due to the covalently closed structures and are widely distributed (Liu et al., 2022b). Studies have shown that circRNAs are widely involved in various physiological and pathological processes, including serving as important modulators of cancer pathogenesis (Wang et al., 2022). CircRNAs participate in tumor development mainly by regulating protein levels via binding to miRNAs (Wang M. et al., 2021). As noted above, VEGFA is an important regulatory protein and plays key roles in cancer pathogenesis (Holmes and Zachary, 2005; Takahashi and Shibuya, 2005; Matsumoto and Ema, 2014; Apte et al., 2019). CircRNAs have been illustrated to regulate VEGFA by binding to different miRNAs (Meng et al., 2019; Dai et al., 2020; Gao S. et al., 2020; Lu et al., 2020). In this review, we will summarize the origin and functional mechanisms of VEGFA and circRNAs, and explore the role of circRNAs in cancer pathogenesis through the regulation of VEGFA.

## VEGFA overview

### VEGFA and receptors

VEGFA exists in most body cells and has many variants as a result of alternative splicing. These variants include VEGFA121, VEGFA145, VEGFA148, VEGFA165, VEGFA189 and VEGFA206 (Koch and Claesson-Welsh, 2012). Generally, VEGFA refers to VEGFA165. VEGFA variants bind to different VEGF receptors and thus have different functions (Koch and Claesson-Welsh, 2012).

**FIGURE 1:**
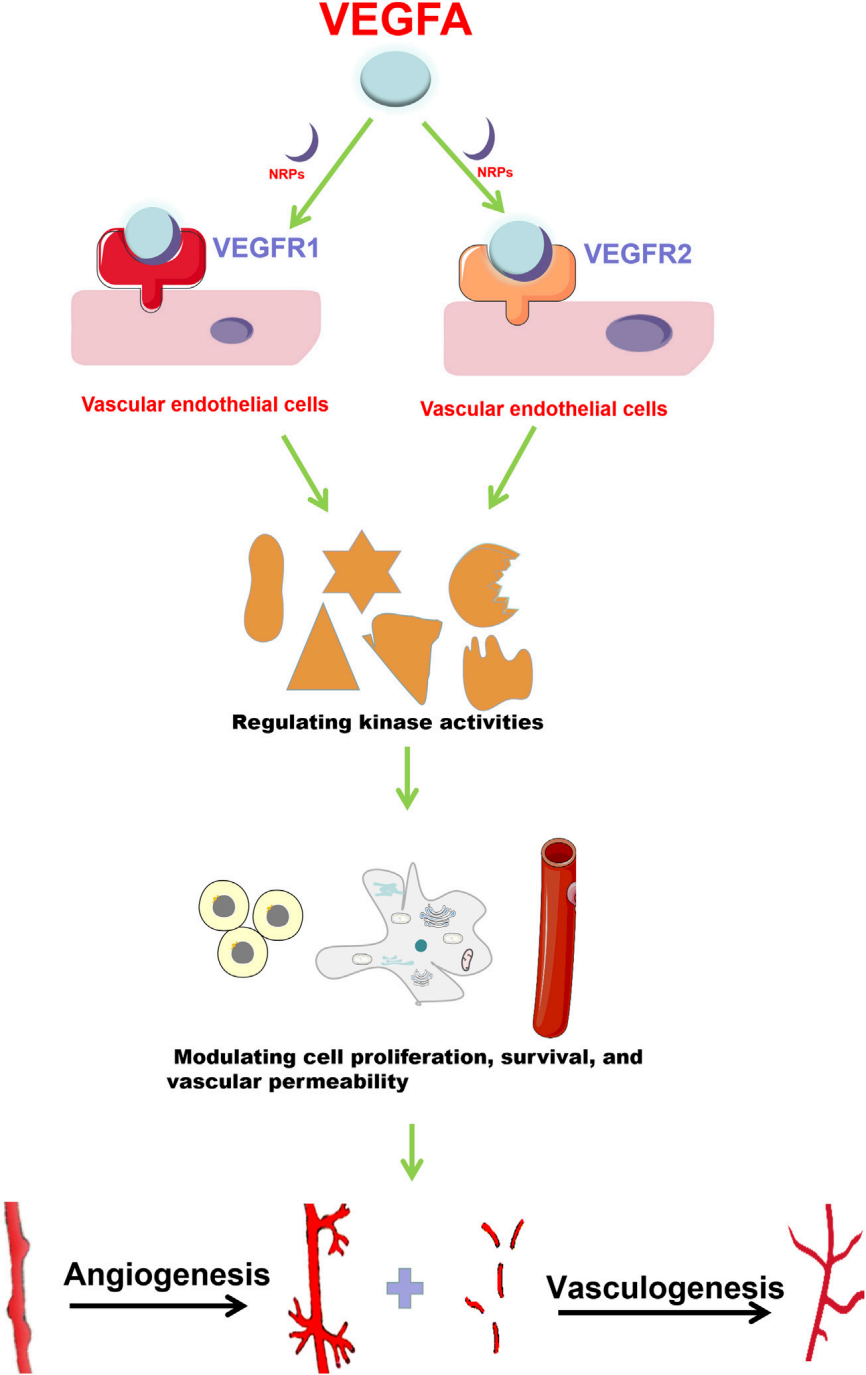
VEGFA promotes angiogenesis and vasculogenesis. VEGFA mainly functions through binding to VEGFR1 and VEGFR2 with the help of NRPs. In vascular endothelial cells, VEGFA interacts with VEGFR1 and VEGFR2 to regulate downstream target activities, and then promotes cell proliferation, survival and vascular permeability, which finally enhances angiogenesis and vasculogenesis.

VEGFA functions by a series of signaling pathways that are mediated by various receptors (Goel and Mercurio, 2013). Receptor Tyrosine kinases (RTK) (VEGFR1, VEGFR2 and VEGFR3) are classical VEGF receptors (Kowanetz and Ferrara, 2006). There are also non-tyrosine kinase receptors (neuropilins, NRPs), which have been revealed to be co-receptors for the RTKs (Soker et al., 1998). The distribution of these receptors is tissue- and cell-specific (Matsumoto and Ema, 2014). VEGFR1 and VEGFR2 are mainly expressed in vascular endothelial cells (ECs) (Carrillo de Santa Pau et al., 2009) (Figure 1), while VEGFR3 is mostly distributed in lymphatic ECs and regulates the generation of lymphangions (Zhang Y. et al., 2014). VEGFA can bind to VEGFR1 or VEGFR2 depending on the type of cell and specific function (s) (Takahashi and Shibuya, 2005). Compared with VEGFR2, VEGFR1 has a stronger binding affinity towards VEGFA, but weaker tyrosine phosphorylation activity (Koch and Claesson-Welsh, 2012). VEGFR2 plays a dominant role in VEGF-mediated angiogenesis and in VEGF signaling in ECs (Kowanetz and Ferrara, 2006).

### VEGFA in cancer pathogenesis

VEGFA is distributed in almost all cell types of tumor tissues, such as cancer cells, tumor-associated macrophages and endothelial cells (Holmes and Zachary, 2005), implying its broad role in cancer development. VEGFA participates in tumorigenesis through different mechanisms, such as regulating angiogenesis and vascular permeability (Senger, 2010), affecting immune cell function (Hansen et al., 2012), and modulating fibroblast function in the cancer stroma (Yaqoob et al., 2012). Moreover, VEGFA can promote epithelial-mesenchymal transition (EMT) and metastasis to influence cancer progression (Kim et al., 2017). High expression of VEGFA indicates a poorer prognosis (Mousa et al., 2015) and may aggravate the development of malignant cancer (Ferroni et al., 2005).

Angiogenesis refers to the development of new blood vessels from existing capillaries or posterior capillaries (Carmeliet and Jain, 2000). Angiogenesis occurs in normal physiological processes such as fetal development, the menstrual cycle and wound closure. Angiogenesis also occurs in pathological processes including the formation of many solid cancers, where it plays a vital role in tumor invasion and metastasis (Bagnasco et al., 2012). VEGFA promotes angiogenesis and vascular permeability through binding to VEGFR1/2 (Senger, 2010; Matsumoto and Ema, 2014). The tumor microenvironment is important for cancer progression as it provides essential resources for cancer cells, such as oxygen, nutrients, and survival factors (Hansen et al., 2012). Tumors can create a special microenvironment to escape monitoring by the immune system, thus promoting cancer development (Coussens and Werb, 2002; Balkwill and Coussens, 2004). VEGFA can function as a chemoattractant for immune cells present in the microenvironment and affect their infiltration into tumors (Hansen et al., 2012).

## CircRNA action mechanisms

**FIGURE 2:**
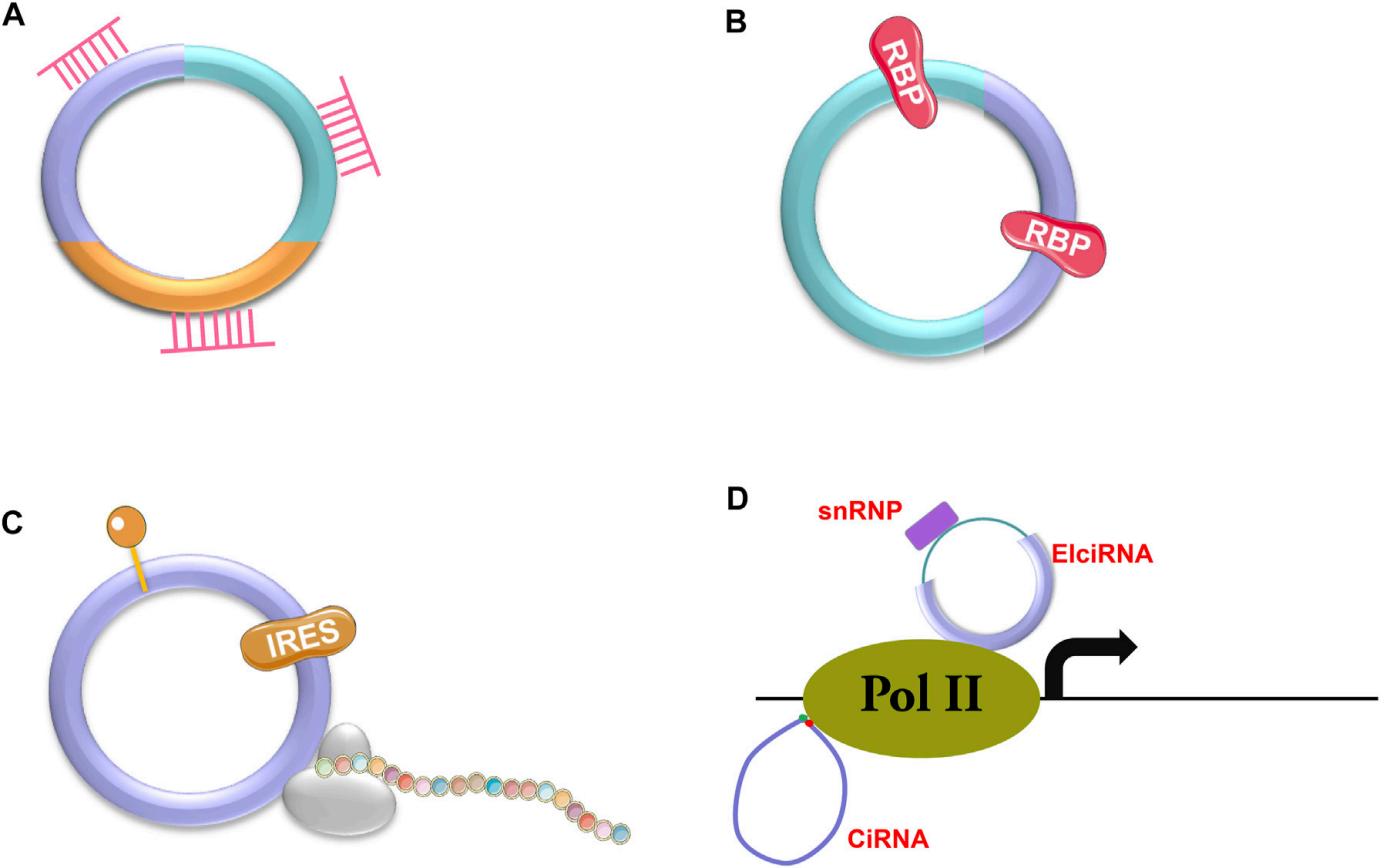
Action mechanisms of circRNAs. CircRNAs can exert their functions through different mechanisms, such as binding to miRNAs (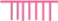) **(A)** or RBPs (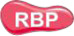) **(B)**, encoding proteins with the help of internal ribosome entry site (IRES, 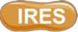) or m6A-modification (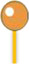) **(C)**, and modulating the transcription of parental genes (ciRNAs and ElciRNAs) **(D)**.

There are three types of circRNAs, including exonic circRNAs (ecircRNAs or ecRNAs) (Zhang X. O. et al., 2014; Zhang et al., 2020), exon-intron circRNAs (EIciRNAs) (Li et al., 2015) and circular intronic RNAs (ciRNAs) (Zhang et al., 2013). Most circRNAs are ecRNAs (Hansen et al., 2013; Memczak et al., 2013), which are mainly located in the cytosol. The other two types of circRNAs localize in the nucleus as they cannot be transported into the cytosol due to the presence of intron sequences (Zhang et al., 2013). CircRNAs have various functions depending on the pathways that they are involved in (Figure 2), such as sponging microRNAs (miRNAs) or RNA binding proteins (RBPs) (Abdelmohsen et al., 2017; Yang et al., 2017; Wang S. et al., 2019; Kong et al., 2020; Ao and Liu, 2022; Liu et al., 2022d) (Figures 2A, B), being templates for protein translation (Yang et al., 2018; Liang et al., 2019) (Figure 2C), or regulating gene expression (Li et al., 2015) (Figure 2D). According to current discoveries, most circRNAs function through their interaction with miRNAs.

Circular RNA nuclear factor IX (circNFIX) facilitates hepatocellular carcinoma (HCC) development via targeting the miR-3064-5p-HMGA2 axis (Xiao et al., 2021). CircRNA Forkhead box O3 (circFoxo3) can inhibit breast cancer by directly binding to the murine double minute 2 protein (Du et al., 2017). Circular F-box and WD repeat domain containing 7 (circFBXW7) encodes a 21-kDa functional protein that has been revealed to suppress the progression of glioma (Yang et al., 2018).

## CircRNA-VEGFA regulatory mechanisms in cancer development

**FIGURE 3:**
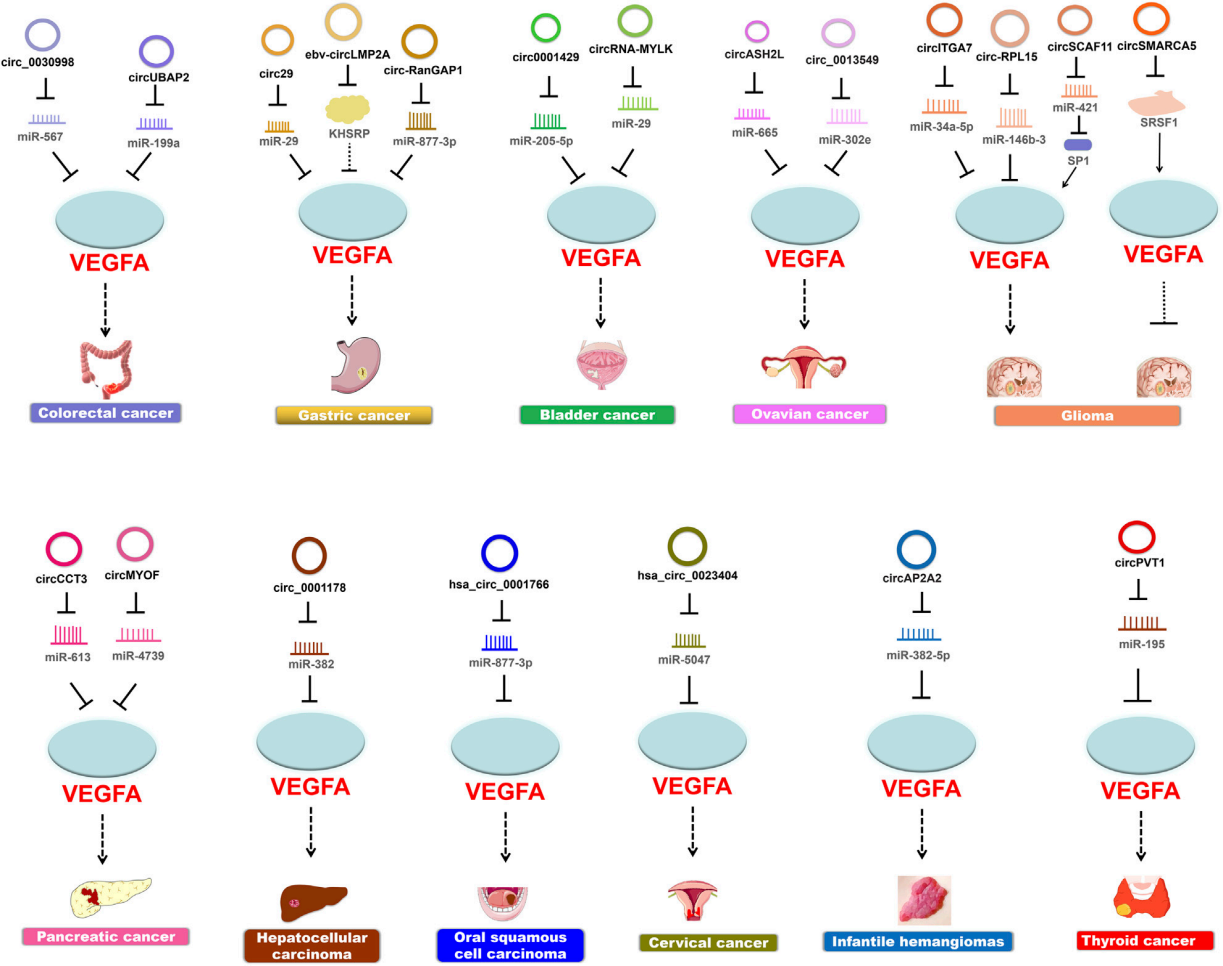
CircRNAs modulate VEGFA to regulate cancer pathogenesis. Most circRNAs upregulate the expression of VEGFA and thereby promote cancer progression.

Guided by the biological function of VEGFA and the expansive role of circRNAs, many findings have demonstrated the regulatory pathways that circRNAs participate in cancer pathogenesis through modulating VEGFA activities (Table 1; Figure 3).

**TABLE 1:** CircRNA---VEGFA axis in cancers.

Cancer types	CircRNA	Sample	Function	Pathway	References
Colorectal cancer(CRC)	Circ_0030998	CRC tissue and cell lines	Promoting cell proliferation and angiogenesis	miR-567-VEGFA	Jin et al. (2021)
	circUBAP2	CRC tissue and cell lines	Promoting cell proliferation, invasion and migration	miR-199a-VEGFA	Dai et al. (2020)
Gastric cancer (GC)	circ29	GC tissues and cell lines	Promoting cell proliferation, invasion, and tube formation	miR-29-VEGFA	Li et al. (2021)
	ebv-circLMP2A	EBVaGC tissues and cells	Promoting tube formation and migration	KHSRP-VEGFA/VHL/HIF1α/	Du et al. (2022)
	circ-RanGAP1	Plasma of GC patients, GC tissues and cells	Facilitating the growth and metastasis of GC cells, promoting tube formation	miR-877-3p-VEGFA	Lu et al. (2020)
Bladder cancer	Circ0001429	Bladder cancer cell	Facilitating the proliferation and metastasis, decreasing cell apoptosis	miR-205-5p--VEGFA	Cao et al. (2019)
	circRNA-MYLK	Bladder cancer cells and tissues	Enhancing cell proliferation, migration, invasion of BC cells, accelerating tube formation of HUVECs and promoting cytoskeleton rearrangment	miR-29a-VEGFA- VEGFR2 -Ras/ERK	Zhong et al. (2017)
Ovarian cancer	circASH2L	Ovarian cancer tissues and cells	Promoting the ovarian cancer cell proliferation and invasion, and repressing tumor xenograft angiogenesis and lymphangiogenesis	miR-665-VEGFA	Chen et al. (2020b)
	circ_0013549	Ovarian cancer tissues and cells	Enhancing ovarian cancer cell viability, invasion and migration ability	miR-302e-VEGFA	Wang et al. (2019a)
Glioma	circITGA7	Glioma tissues and cell lines	Promoting glioma cell growth, invasion and migration	miR-34a-5p-VEGFA	Qi et al. (2021)
	circ-RPL15	Glioma tissues and cell lines	Promoting glioma cell viability and migratory ability	miR-146b-3-VEGFA	Wang et al. (2020)
	circSCAF11	Glioma tissues and cell lines	Promoting the tumor cell proliferation and invasion, triggering cell cycle arrest, and inhibiting tumor tissue growth	miR-421-SP1-VEGFA	Meng et al. (2019)
Glioblastoma (GBM)	CircSMARCA5	GBM tissues	Inhibiting GBM progression by suppressing pro-angiogenic function of VEGFA via downregulating SRSF1	SRSF1-VEGFA	Barbagallo et al. (2019)
Pancreatic Cancer	circCCT3	PC tissues and cells	Promoting PC tissue growth and enhancing the migration and invasion of PC cell lines	miR-613-VEGFA-VEGFR2	Hou et al. (2021)
Pancreatic ductal adenocarcinoma (PDA)	circMYOF	PDA tumor and cell lines	Promoting PDA tumor growth and PDA cell proliferation and metastasis	miR-4739-VEGFA	Zheng et al. (2021)
				miR-4739-PI3K/AKT	
Hepatocellular carcinoma (HCC)	circ_0001178	HCC tissues and cell lines	Promoting HCC cell proliferation, migration and invasion ability, enhancing HCC cell apoptosis and HCC cell cycle	miR-382-VEGFA	Gao et al. (2020b)
Oral squamous cell carcinoma (OSCC)	hsa_circ_0001766	OSCC tissues and cell lines	Enhancing OSCC cell proliferation and tumor initiation	miR-877-3p-VEGFA	Shao et al. (2020)
Cervical cancer	hsa_circ_0023404	Cervical cancer tissues and cell lines	Enhancing the cell invasion and lymphatic vessel formation, inhibiting autophagy treated by cisplatin	miR-5047-VEGFA	Guo et al. (2019)
Infantile hemangiomas	CircAP2A2	IH tissues and cell lines	Promoting hemangioma cell proliferation, invasion, and migration	miR-382-5p-VEGFA	Yuan et al. (2020)
Thyroid cancer (TC)	CircPVT1	PTC tissues and cell lines	Promoting PTC cell proliferation, migration, and invasion ability	miR-195-VEGFA	Zeng et al. (2021)

### Colorectal cancer

Colorectal cancer (CRC) is one of the most common and lethal malignancies (Bray et al., 2018). For advanced CRC, the 5-year survival rate is less than 20% (Siegel et al., 2017). Even with improvement in treatment, the survival rate of CRC patients has not been improved significantly. Additional information on the pathogenesis of CRC will provide theoretical strategies and foundations towards improved clinical treatment. VEGFA has been shown to reduce the survival rates of CRC patients (Goos et al., 2016). Recently, circRNAs have been found to contribute to the progression of CRC with some of them functioning through their role in regulating VEGFA expression (Chen C. et al., 2020; Dai et al., 2020; Jin et al., 2021).

Jin et al. performed a microarray-based expression profile analysis of CRC tissues and neighboring normal tissues (Jin et al., 2021). Comparative gene expression analysis identified a series of circRNAs with altered expression. Circ_0030998 was noted to exhibit the most significant change in expression in CRC tissues and cells, where circ_0030998 levels were elevated (Jin et al., 2021). Kaplan–Meier survival analysis showed that high expression levels of circ_0030998 negatively correlated with survival rates, implying a poor prognosis. Univariate survival analysis and Multivariate Cox regression analysis revealed that circ_0030998 expression level was an independent prognostic factor for CRC. Circ_0030998 promoted the proliferation of CRC cells and angiogenesis of human umbilical vein endothelial cells (HUVECs). On the other hand, circ_0030998 downregulation inhibited CRC cell proliferation in nude mice. Bioinformatics analyses, luciferase reporter assays and RNA immunoprecipitation (RIP) assays confirmed that circ_0030998 interacted with miR-567 and miR-567 downregulation increased CRC cell proliferation and the formation of tube-like structure in HUVECs. MiR-567 mimics rescued the effect of circ_0030998 on cell proliferation and angiogenesis, suggesting that circ_0030998 might function through sponging miR-567. Bioinformatics analyses predicted that miR-567 might target VEGFA to regulate CRC cell proliferation and angiogenesis (Jin et al., 2021). VEGFA levels were significantly increased in CRC cell lines and negatively associated with miR-567 levels in CRC tissues. Luciferase reporter assays showed that miR-567 could interact with VEGFA. VEGFA was also demonstrated to promote the CRC cell cycle and tube-like structure formation in HUVECs, consistent with the effect of inhibiting miR-567. Circ_0030998 knockdown decreased VEGFA levels, while circ_0030998 overexpression positively regulated VEGFA levels. Knockdown of VEGFA suppressed the promoting effect of circ_0030998 on CRC cell cycle progression and tube-like structure formation (Jin et al., 2021). All findings indicate that circ_0030998 might regulate CRC progression by promoting cell proliferation and angiogenesis via the miR-567-VEGFA axis (Jin et al., 2021).

CircRNA UBAP2 (circUBAP2, Ubiquitin Associated Protein 2) has been implicated as an important regulator in multiple malignancies (Dai et al., 2020). The mechanism of how circUBAP2 regulates CRC progression through VEGFA has also been clarified (Dai et al., 2020). CircUBAP2 expression was upregulated *in vivo* and *in vitro*. CircUBAP2 depletion suppressed CRC development by inhibiting cell proliferation, invasion and migration (Dai et al., 2020). MiR-199a was identified as the target of circUBAP2. MiR-199a downregulation rescued the inhibitory effect of circUBAP2 on CRC development. Furthermore, VEGFA was validated as the target of miR-199a. CircUBAP2 overexpression increased VEGFA levels and decreased miR-199a levels. VEGFA overexpression promoted tumorigenesis that was attenuated when circUBAP2 was downregulated or miR-199a overexpressed (Dai et al., 2020). All findings demonstrate that circUBAP2 promotes CRC progression by modulating VEGFA via sponging miR-199a (Dai et al., 2020).

### Gastric cancer

Gastric cancer (GC) is a cancer of the digestive system, which is lethal and results in high mortality rates (Li et al., 2021). Many factors influence GC development, including VEGFA (Li et al., 2021). CirRNAs have been found to participate in GC pathogenesis through regulating VEGFA (Lu et al., 2020; Li et al., 2021; Du et al., 2022).

Hsa-circ_0044366 is generated from the exons of the *ATP5G1* gene, and termed circ29 (Li et al., 2021). In an RNA microarray analysis, expression of circ29 was elevated in the plasma of GC patients. In other GC samples, circ29 was also upregulated. Circ29 overexpression could promote tumor development *in vivo*. In plasma, circ29 is mainly distributed in the exosomes (Li et al., 2021). Exosomal circ29 was extracted from GC cells transfected with ovexpression-circ29/si-cir29 vectors and added into HUVECs. Exosomal circ29 from ovexpressed-circ29 GC cells significantly promoted the proliferation, invasion and tube formation of HUVECs, proposing the oncogenic role of circ29 (Li et al., 2021). The tumor promoting effect of circ29 could be repressed by miR-29. VEGFA was further validate as the downstream target of miR-29. Further experiments confirmed that circ29 regulated VEGFA by sponging miR-29. In brief, circ29 plays an oncogenic role in GC development via the miR-29-VEGFA axis (Li et al., 2021).

Epstein-Barr virus (EBV) is one of the most studied human oncogenic viruses and has been revealed to infect over 90% of adults worldwide (Du et al., 2022). Some individuals infected with EBV might develop EBV-associated cancers (Thorley-Lawson, 2001), such as GC (Wong et al., 2022). This type of GC is referred to as EBVaGC, the largest subtype of EBV-associated cancers. Unlike normal GC, EBVaGC has its own unique molecular mechanism and clinical characteristics (Cancer Genome Atlas Research Network, 2014). Ebv-circLMP2A (Latent Membrane Protein 2A) was highly expressed in EBVaGC and had prognostic potential (Du et al., 2022). Expression of ebv-circLMP2A was positively correlated with angiogenesis-related markers such as VEGFA, HIF1α (alpha subunit of transcription factor hypoxia-inducible factor-1), and microvessel density in EBVaGC tissues. In hypoxia-treated HUVECs, ebv-circLMP2A was upregulated and promoted tube formation and migration. Further analyses demonstrated that ebv-circLMP2A could facilitate EBVaGC pathogenesis through promoting angiogenesis via the KHSRP-VHL/HIF1α/VEGFA axis under hypoxic conditions (Du et al., 2022).

In the circRNA microarray analyses conducted on GC tissues and exosomes from the preoperative plasma of GC patients, hsa_circ_0063526 was found to be significantly upregulated (Lu et al., 2020). Hsa_circ_0063526 was identified to be an ecRNA transcribed from the *RanGAP1* (Ran GTPase Activating Protein 1) gene and was also referred to as circ-RanGAP1. High levels of circ-RanGAP1 were positively related with advanced tumor, node, metastasis (TNM) stages and high lymph node metastases. Further analysis proposed that high circ-RanGAP1 level implied poor overall survival and was an independent GC prognostic factor. The use of circRanGAP1 expression and TNM stage in combination might be more sensitive than either circRanGAP1 or TNM stage alone to determine GC prognosis. Circ-RanGAP1 knockdown suppressed GC cell invasion and migration, while circ-RanGAP1 overexpression had the opposite effect, suggesting an oncogenic role. In xenograft tumor models, circ-RanGAP1 facilitated tumor growth and metastasis (Lu et al., 2020). Further analyses validated that circ-RanGAP1 could sponge miR-877-3p. MiR-877-3p overexpression restrained the role of circ-RanGAP1 in GC progression. VEGFA was found to be the downstream target of miR-877-3p and they were negatively associated. VEGFA expression was increased in GC tissues. VEGFA knockdown suppressed the proliferation, invasion and migration of GC cell lines, while VEGFA overexpression promoted the formation of capillary-like tubular structures. In GC samples, VEGFA and circ-RanGAP1 levels were positively correlated. The combination of circ-RanGAP1 and VEGFA expression was shown to have superior and more accurate prognostic potential for GC patients. All results demonstrated that circ-RanGAP1 might aggravate GC symptoms by upregulating VEGFA via targeting miR-877-3p (Lu et al., 2020).

### Bladder cancer

Bladder cancer is common in the elderly, especially in males. Most patients with bladder cancer can be diagnosed at an early stage. However, prognosis is poor and the recurrence rate for bladder cancer is quite high. CircRNAs have prognostic value in bladder cancer and can regulate VEGFA expression to affect the formation of bladder cancer (Zhong et al., 2017; Cao et al., 2019).

Circ0001429 expression was remarkably upregulated in bladder cancer tissues based on data from a microarray-based study (Cao et al., 2019). Circ0001429 facilitated the proliferation and metastasis of bladder cancer cells *in vitro*, and decreased cell apoptosis. Circ0001429 knockdown inhibited the formation of tumors in the bladder and prevented cancer from metastasizing to the lung. VEGFA expression was also elevated in bladder cancer tissues. More experiments validated that circ0001429 could bind to miR-205-5p and miR-205-5p could interact with VEGFA. MiR-205-5p suppressed bladder cancer cell viability and mobility, and enhanced apoptosis (Cao et al., 2019). MiR-205-5p overexpression inhibited the effect of circ0001429 on bladder cancer. To sum up, circ0001429 may sponge miR-205-5p to increase the levels of VEGFA, thereby exacerbating bladder cancer symptoms (Cao et al., 2019).

Zhong et al. carried out a microarray-based analysis to screen circRNA and mRNA expression profiles using bladder cancer samples (Zhong et al., 2017). Among all circRNAs and mRNAs, CircRNA-MYLK (myosin light chain kinase) and four VEGFA variants were shown to be significantly upregulated. Expression of circRNA-MYLK was positively correlated with that of VEGFA (Zhong et al., 2017). Overexpression of circRNA-MYLK enhanced cell proliferation, migration, invasion of bladder cancer cells, accelerated tube formation in HUVECs and promoted cytoskeleton rearrangement (Zhong et al., 2017). Survival curve analyses revealed that high circRNA-MYLK levels were positively associated with poor prognosis. CircRNA-MYLK upregulation could promote tumor growth of xenografts as well as metastasis. Subsequent analyses showed that circRNA-MYLK might bind to miR-29a to regulate the expression of VEGFA (Zhong et al., 2017). VEGFA functioned through binding to VEGFR2 and activated the Ras/ERK signaling pathway (Zhong et al., 2017). This suggests that circRNA-MYLK is an oncogene and might function through the activation VEGFA/VEGFR2 and the Ras/ERK signaling pathway via targeting miR-29a (Zhong et al., 2017).

### Ovarian cancer

Ovarian cancer is a key cancer of the female reproductive system and causes a high number of deaths. With the progress in clinical treatment for ovarian cancer, there is a slight decrease in the number of deaths from ovarian cancer in recent years. CircRNAs are found to participate in ovarian cancer development (Wang L. L. et al., 2019; Chen J. et al., 2020).

CircASH2L (histone methyltransferase complex subunit ASH2) was visibly upregulated in ovarian cancer tissues and cells (Chen J. et al., 2020). Knockdown of circASH2L attenuated ovarian cancer cell proliferation and invasion. Moreover, knockdown of circASH2L repressed tumor xenograft angiogenesis and lymphangiogenesis through suppression of VEGFA (Chen J. et al., 2020). CircASH2L and VEGFA levels were positively associated with each other in ovarian cancer tissues. Overexpression of VEGFA rescued the effect of circASH2L knockdown on tumorigenesis by upregulating VEGFR2 and VEGFR3. Further analyses confirmed that circASH2L might regulate VEGFA by sponging miR-665 (Chen J. et al., 2020). Therefore, circASH2L might function as an oncogene to promote the pathogenesis of ovarian cancer through the miR-665-VEGFA axis (Chen J. et al., 2020).

Circ_0013549 is produced from the *RhoC* (Ras Homolog Family Member C) gene, an oncogene in ovarian cancer (Wang L. L. et al., 2019). Thus, circ_0013549 is referred to as circRhoC (Wang L. L. et al., 2019). CircRhoC was upregulated in ovarian cancer tissues and cells. CircRhoC overexpression enhanced ovarian cancer cell viability, invasion and migration, while the knockdown of circRhoC had the opposite effects (Wang L. L. et al., 2019). CircRhoC was reported to promote VEGFA expression. MiR-302e was the downstream target of circRhoC and directly regulated VEGFA expression. CircRhoC might promote ovarian cancer development by upregulating miR-302e via targeting VEGFA. Moreover, circRhoC was also found to directly bind to VEGFA (Wang L. L. et al., 2019). Therefore, circRhoC might regulate ovarian cancer through two different pathways (Wang L. L. et al., 2019).

### Glioma

Glioma is the most common primary intracranial tumor and can be divided into different subtypes. Glioma has high morbidity, recurrence, and mortality rates, especially for anaplastic astrocytoma and glioblastoma (Louis et al., 2016). Glioblastoma (GBM) is the fourth grade glioma with the highest degree of malignancy and the worst prognosis (Global Burden of Disease Cancer Collaboration et al., 2019; Khani et al., 2019).

CircITGA7 has been revealed to be involved in tumorigenesis (Li et al., 2018). Qi et al. explored the role of circITGA7 in glioma and noted that circITGA7 expression was remarkably increased in glioma tissues and cells (Qi et al., 2021). CircITGA7 knockdown inhibited glioma cell proliferation and metastasis. CircITGA7 was found to sponge miR-34a-5p and regulate its activity. MiR-34a-5p targeted VEGFA to decrease VEGFA expression (Qi et al., 2021). MiR-34a-5p downregulation promoted glioma cell growth, invasion and migration but VEGFA knockdown reversed the effect of miR-34a-5p downregulation on tumorigenesis. All data suggest that circITGA7 promotes glioma tumorigenesis by the miR-34a-5p-VEGFA axis (Qi et al., 2021).

Overexpression of circ-RPL15 was observed in glioma tissues and cell lines (Wang et al., 2020). Knockdown of circ-RPL15 decreased glioma cell viability and migratory ability. Circ-RPL15 was revealed to mainly localize in the cytoplasm (Wang et al., 2020). Further experiments confirmed that circ-RPL15 could bind to miR-146b-3. MiR-146b-3 expression was decreased in glioma tissues and cell lines. VEGFA was positively correlated with circPRL15 and negatively correlated with miR-146b-3. Overexpression of miR-146b-3p reduced the level of VEGFA (Wang et al., 2020). In general, circ-RPL15 might promote glioma progression by suppressing miR-146b-3 and thus upregulating VEGFA activity (Wang et al., 2020).

CircSCAF11 is generated from the exons of the *SCAF11* (SR-Related CTD Associated Factor 11) gene. CircSCAF11 levels were elevated in glioma tissues and cell lines (Meng et al., 2019). Overexpression of circSCAF11 was positively correlated with poor clinical prognosis of glioma patients (Meng et al., 2019). Downregulation of circSCAF11 repressed tumor cell proliferation and invasion, triggered cell cycle arrest, and inhibited tumor tissue growth. Further analysis revealed that miR-421 was the downstream target of circSCAF11 and that the SP1 transcription factor was the target of miR-421. CircSCAF11 might promote SP1 expression by suppressing miR-421 expression (Meng et al., 2019). Furthermore, as a transcription factor involved in the transformation of normal cells into cells with malignant properties, SP1 was shown to bind to the VEGFA promoter region and activate *VEGFA* transcription. All results indicate that the miR-421-SP1-VEGFA axis may be driven by circSCAF11 in the development of glioma (Meng et al., 2019).

CircSMARCA5 has been reported to participate in GBM development (Barbagallo et al., 2018). CircSMARCA5 expression was decreased in GBM tissues proposing that circSMARCA5 levels are negatively correlated with the overall survival of GBM patients (Barbagallo et al., 2019). CircSMARCA5 was predicted to act as a sponge for the serine and arginine rich splicing factor 1 (SRSF1) through more than seven evolutionarily conserved binding sites and their physical interaction has since been experimentally validated (Barbagallo et al., 2019). This interaction was thought to regulate the switch between pro- and anti-angiogenic isoforms produced by the splicing of VEGFA pre-mRNA in GBM cells. The ratio of pro-angiogenic to anti-angiogenic VEGFA mRNA isoforms was elevated in GBM cells compared with controls, while the ratio was reduced in GBM cells with overexpressed circSMARCA5. Thus, circSMARCA5 might inhibit GBM progression by suppressing the pro-angiogenic function of VEGFA via downregulating SRSF1 (Barbagallo et al., 2019).

### Pancreatic cancer

Pancreatic cancer (PC) is a lethal malignancy that arises from the cells in the pancreatic duct. The symptoms of PC are usually not apparent until it has developed into the later stages and spread to other organs. Its 5-year relative survival rate is only 9%. Pancreatic ductal adenocarcinoma (PDA) and its subtypes are the most common PCs, accounting for 85%–90% of PCs. Fully comprehending the mechanisms that drive PC progression is essential to develop effective diagnostic and treatment strategies.

Chaperonin containing TCP1 subunit 3 protein (CCT3) plays important roles in tumorigenesis (Temiz et al., 2021). CircCCT3 is produced from the *CCT3* gene by back-splicing (Lv et al., 2020). While the role of circCCT3 in other cancers has been elucidated (Lv et al., 2020), Hou et al. reported that circCCT3 expression was significantly upregulated in PC tissues and cells (Hou et al., 2021). CircCCT3 overexpression promoted PC tissue growth and enhanced PC cell migration and invasion. Further to this, elevated circCCT3 levels signified poor overall survival of PC patients but circCCT3 level was an independent PC prognostic factor. CircCCT3 downregulation suppressed tumor growth in nude mice. This effects of circCCT3 on PC progression could be attenuated by the addition of a miR-613 mimic. MiR-613 was the target of circCCT3 and further analyses revealed the direct interaction between miR-613 and VEGFA (Hou et al., 2021). CircCCT3 overexpression promoted the expression of VEGFA and VEGFR2, while circCCT3 knockdown decreased their levels (Hou et al., 2021). In conclusion, circCCT3 has prognostic potential for PC and might deteriorate PC symptoms via the miR-613-VEGFA-VEGFR2 pathway (Hou et al., 2021).

Reprogrammed glucose metabolism is a typical feature of pancreatic malignancy (Du et al., 2020), including PDA. RNA sequencing of low glucose-treated PDA cells identified a novel circRNA, circMYOF (Zheng et al., 2021). CircMYOF was located at the cytoplasm and was upregulated in PDA clinical tissues and cell lines. Overexpression of circMYOF led to PDA tumor growth and PDA cell proliferation and metastasis, while circMYOF knockdown had the opposite effect (Zheng et al., 2021). Upregulation of circMYOF promoted glucose and lactate production. The PI3K/AKT pathway is very important for glycolysis. Increased PI3K and phospho-AKT (Ser473) levels were observed in PDA cells overexpressing circMYOF. Inhibition of PI3K/AKT repressed effect of circMYOF overexpression on promoting PDA. Moreover, circMYOF was a sponge of miR-4739 and circMYOF-miR-4739 functioned through targeting VEGFA or PI3K/AKT pathway (Zheng et al., 2021). Taken together, circMYOF might promote PDA progression by two independent pathways (Zheng et al., 2021).

### Hepatocellular carcinoma

Hepatocellular carcinoma (HCC) is a form of liver cancer and usually occurs in people with chronic liver diseases (Gao S. et al., 2020). Early diagnosis of HCC is very challenging and the malignant form of HCC causes a significant number of deaths worldwide. Circ_0001178 was shown to enhance the pathogenesis of HCC, whereby circ_0001178 levels were remarkably increased in HCC tissues and cell lines. Circ_0001178 knockdown repressed HCC cell proliferation, migration and invasion, induced HCC cell apoptosis, and blocked the HCC cell cycle (Gao S. et al., 2020). Moreover, circ_0001178 knockdown also inhibited tumor growth *in vivo*. Analyses revealed the interaction between miR-382 and circ_0001178 with circ_0001178 inhibiting miR-382 activity. Experiments demonstrated that VEGFA was the direct target of miR-382. VEGFA levels were suppressed by miR-382 but promoted by circ_0001178. Therefore, circ_0001178 may promote HCC progression via the miR-382-VEGFA axis (Gao S. et al., 2020).

### Oral squamous cell carcinoma

Oral squamous cell carcinoma (OSCC) mainly occurs in the mouth. It is the most malignant and damaging tumor in the head and neck region (Shao et al., 2020). The lymph nodes in OSCC patients are highly metastatic leading to poor prognosis. Shao et al. carried out high-throughput sequencing of OSCC tissues and more than 100 differentially expressed circRNAs were identified (Shao et al., 2020). One of the upregulated circRNAs, hsa_circ_0001766, was selected for further analysis. Hsa_circ_0001766 is a novel circRNA produced from the *PDIA4* (protein disulfide-isomerase A4) gene. Bioinformatics analyses showed that hsa_circ_0001766 might sponge miR-877-3p and that VEGFA was the direct target of miR-877-3p (Shao et al., 2020). VEGFA was reported to enhance OSCC cell proliferation and tumor initiation (Gao P. et al., 2020). High expression levels of VEGFA were related to significantly poor prognosis in OSCC (Sales et al., 2016). Hsa_circ_0001766 induced OSCC cell proliferation, while hsa_circ_0001766 knockdown inhibited the expression of VEGFA and repressed cell proliferation. MiR-877-3p inhibitor rescued the inhibitory effect of hsa_circ_0001766 downregulation on cell proliferation. In conclusion, hsa_circ_0001766 may promote OSCC pathogenesis through the miR-877-3p-VEGFA axis (Shao et al., 2020).

### Cervical cancer

Cervical cancer starts in the cells of the cervix and is the fourth most common cancer among women globally. Cervical cancer is a leading cause of cancer-related mortality due to metastasis and drug resistance (Peng et al., 2016). Chemotherapy is one of the commonly used clinical treatments for cervical cancer (Feng et al., 2017). Guo et al. explored the mechanism of hsa_circ_0023404 in modulating cervical cancer metastasis and chemoresistance (Guo et al., 2019). Lymphangiogenesis, the process that leads to the formation of new lymphatic vessels from pre-existing vessels, is an important route to tumorigenesis and metastasis (Sleeman and Thiele, 2009). Downregulation of hsa_circ_0023404 in cervical cancer cell lines restrained cell invasion and lymphatic vessel formation, suggesting that hsa_circ_0023404 stimulates metastasis (Guo et al., 2019). Hsa_circ_0023404 was found to sponge miR-5047. Knockdown of miR-5047 inhibited the effect of hsa_circ_0023404 downregulation on metastasis. Hsa_circ_0023404 knockdown and a miR-5047 mimic reduced VEGFA expression (Guo et al., 2019). Hsa_circ_0023404 and VEGFA were upregulated in cervical cancer tissues and were positively correlated with cervical cancer development, while miR-5047 silencing had the opposite role (Guo et al., 2019). Further analyses showed that VEGFA was an important downstream target for hsa_circ_0023404/miR-5047-mediated metastasis. In cisplatin-treated cervical cancer cells, hsa_circ_0023404 knockdown promoted autophagy, which could be abolished by the addition of an autophagy inhibitor (Guo et al., 2019). Apoptosis rates were increased in hsa_circ_0023404 knockdown cells treated with 2 μg/mL cisplatin. In general, hsa_circ_0023404 plays an important role in the progression of cervical cancer via the miR-5047-VEGFA axis (Guo et al., 2019).

### Infantile hemangioma

Hemangioma is a benign tumor originating from cutaneous blood vessels and usually occurs in infants or children (Leaute-Labreze et al., 2017; Yuan et al., 2020). Compared with other tumors, infantile hemangioma (IH) has unique characteristics (Leaute-Labreze et al., 2017). It grows rapidly in infants and recedes in children (Leaute-Labreze et al., 2017). In most cases, IH is harmless and the children do not require treatment. However, around 10% of lesions still require treatment to reduce possible threats (Sans et al., 2009). CircAP2A2 is an ecRNA that localizes in the cytoplasm and is derived from the *AP2A2* gene (Yuan et al., 2020). CircAP2A2 is highly expressed in IH tissues in comparison with normal tissues (Yuan et al., 2020). CircAP2A2 downregulation significantly weakened hemangioma cell proliferation, invasion, and migration. CircAP2A2 interacted with miR-382-5p and their expression levels were negatively correlated (Yuan et al., 2020). VEGFA was confirmed as the direct target of miR-382-5p. CircAP2A2 knockdown reduced the expression of VEGFA, and this could be rescued by the downregulation of miR-382-58. All the results indicate that circAP2A2 may promote IH progression through the miR-382-5p-VEGFA pathway (Yuan et al., 2020).

### Papillary thyroid carcinoma

Thyroid cancer (TC) is an endocrine tumor of low malignancy with good prognosis. Papillary thyroid cancer (PTC) is the most common histological type of TC (Fahiminiya et al., 2016). Typically, PTC patients receive a good prognosis, however, some may undergo lymphatic metastasis, early epidural infiltration or other high-risk complications (Zeng et al., 2021). CircPTV1 was an important regulator of PTC pathogenesis. CircPVT1 levels were increased in PTC tissues and cell lines (Zeng et al., 2021). High circPVT1 levels were positively correlated with tumor growth and metastasis. Silencing of circPVT1 suppressed PTC cell proliferation, migration, and invasion ability as well as inhibited the Wnt/β-catenin signaling pathway (Zeng et al., 2021). MiR-195 was validated to interact with both circPVT1 and VEGFA. VEGFA downregulation repressed Wnt/β-catenin signaling pathway, similar to the circPTV1 knockdown. CircPVT1 was shown to sponge miR-195 and then facilitate the expression of VEGFA to promote PTV progression (Zeng et al., 2021). Therefore, the circPVT1-miR-195-VEGFA axis might be valuable to determine prognosis and therapeutic therapies for PTC (Zeng et al., 2021).

Taken together, the various circRNA-miRNA-VEGFA pathways play important roles in the occurrence and development of different cancers. This raises the possibility of a similar mechanism involved in other cancers such as renal cancer, prostate cancer, etc., which should be further investigated.

## Clinical potential and future perspective

In 2004, the Food and Drug Authority of United States approved the marketing of a drug that can inhibit tumor angiogenesis. This drug, bevacizumab (Avastin), is a recombinant humanized monoclonal antibody. As an anti-cancer agent, bevacizumab binds to VEGFA and blocks its biological activity in promoting tumor angiogenesis. Bevacizumab can be used in the treatment of various cancers (Ferrara and Kerbel, 2005). However, treating cancer patients with only bevacizumab is less effective in most advanced malignancies. VEGFA is a multifunctional protein that plays a critical role in cancer pathogenesis. Hence, further understanding of mechanisms utilized by VEGFA and analyzing its upstream and downstream pathways will help in the development of new drugs and propose more precise and effective combined treatment strategies.

Noncoding RNAs, such as miRNAs and long noncoding RNAs (lncRNAs), have been found to be promising biomarkers for disease diagnosis or prognosis (Liu et al., 2022a; Wang et al., 2022; Zhang et al., 2022; Zhou et al., 2022). Moreover, miRNAs can be targets for the therapeutic drugs. Tiny Locked nucleic acid (LNA)-modified antimiRs, special miRNA inhibitors, have been proven to have effective effects in the treatment of tumors and cardiovascular diseases (Obad et al., 2011; Bernardo et al., 2012; Murphy et al., 2013). The combinatorial RNA therapeutics using the combinations of antimiRs, siRNAs and miRNA mimics have been determined to suppress the proliferation of many lung cancer cell lines (Petrek et al., 2019). Some RNA therapies based on miRNA mimics and anti-microRNA inhibitors are in the clinical phase II or III development stage (Winkle et al., 2021). Compared with miRNAs, the targeted therapies of lncRNAs are not mature, and there are many obstacles. Therefore, lncRNA-based therapeutic therapies have not yet entered clinical practice.

More recently, the clinical value of circRNAs has also been explored. First, circRNAs have several properties, such as high stability, wide distribution, and tissue- and developmental stage-specific expression, which make them ideal biomarkers for diagnosis. Furthermore, expression levels of circRNAs in the blood are sufficiently high, making detection easier (Xu et al., 2017; Zhang et al., 2020; Sun et al., 2021). To date, some circRNAs have been patented for their clinical applications. For example, circRNAs can be used as diagnostic markers of disease or viral infections, as vectors for vaccine research and development, and as markers for forensic identification. Secondly, some circRNAs can produce functional proteins. CircMAPK14 encodes a peptide, which can be used to formulate targeted anti-tumor drugs (Wang L. et al., 2021). Till now, the research on circRNAs has not yet entered the clinical stage, but the value of circRNAs in clinical treatment is highly anticipated. CircRNAs are important upstream regulators of VEGFA. Compared with bevacizumab, circRNAs are highly specific in regulating VEGFA in different cancers (Yuan et al., 2020; Qi et al., 2021). The combined application of bevacizumab and circRNAs might serve as targeted-specific drugs for the treatment of cancers as opposed to the use of broad-spectrum chemotherapeutics. This will greatly improve treatment effectiveness for specific cancers.

Despite the strengths of circRNAs, we conclude that there are still limitations that affect the clinical use. First, the names given to circRNAs are often ambiguous and lack consistency across studies, increasing the difficulty of subsequent research. Recently, however, Chen et al. developed a new nomenclature for naming circRNAs (Chen et al., 2023). They proposed that the names of circRNAs could begin with circ ‘host gene symbol’ followed by the exon and/or intron information. We believe that this method can solve this problem well. Second, the current research and existing patents only focus on the roles of circRNAs in clinical diagnosis or prognosis. Although circRNAs are potential drug targets, there are no directly related findings on its use for clinical therapy. Third, no studies have examined the potential of combining circRNAs with VEGFA targeted drugs. We look forward to in-depth new research that will address these limitations and facilitate the potential clinical application.

## Conclusion

The VEGFA regulatory system is multifunctional and VEGFA is involved in cancer pathogenesis. CircRNAs can modulate the activity of VEGFA mainly by binding to miRNAs. Most circRNAs upregulate VEGFA expression, and high levels of VEGFA exacerbate the symptoms of various cancers. The combined use of circRNAs and VEGFA as cancer therapeutic targets might provide an opportunity for new forms of cancer treatment. However, due to the limitations discussed above, there is still a long way to go for their combined use in clinical treatment.

## Abbreviations

VEGFA, vascular endothelial growth factor-A; circRNA, circular RNAs; RTK, Tyrosine kinase; ECs, endothelial cells; ecRNAs, exonic circRNAs; EIciRNAs, exon-intron circRNAs; ciRNAs, circular intronic RNAs; miRNAs, microRNAs; RBPs, RNA binding proteins; HCC, hepatocellular carcinoma; CRC, colorectal cancer; HUVECs, human umbilical vein endothelial cells; GC, gastric cancer; EBV, Epstein-Barr virus; GBM, glioblastoma; PC, pancreatic cancer; PDA, pancreatic ductal adenocarcinoma; OSCC, oral squamous cell carcinoma; IH, infantile hemangioma; TC, Thyroid cancer; PTC, papillary thyroid cancer; Avastin, Bevacizumab.
